# Premature ageing of lung alveoli and bone marrow cells from *Terc* deficient mice with different telomere lengths

**DOI:** 10.1038/s41598-025-90246-2

**Published:** 2025-02-19

**Authors:** Rosa Guerrero-López, Cristina Manguán-García, Carlos Carrascoso-Rubio, M. Luz Lozano, Marta Toldos-Torres, Laura García-Castro, Rebeca Sánchez-Dominguez, Omaira Alberquilla, Isabel Sánchez-Pérez, Maria Molina-Molina, Juan A. Bueren, Guillermo Guenechea, Rosario Perona, Leandro Sastre

**Affiliations:** 1 Instituto de Investigaciones Biomedicas Sols/Morreale, CSIC-UAM. Arturo Duperier, Madrid, 28029 Spain; 2 Centro de Investigacion Biomedica en Red de Enfermedades Raras (CIBERER), Madrid, 28029 Spain; 3 Instituto de Salud Carlos III, Madrid, 28029 Spain; 4 Hematopoietic Innovative Therapies Division, Centro de Investigaciones Energeticas, Medioambientales y Tecnologicas (CIEMAT)) and Advanced Therapies Unit, Instituto de Investigación Sanitaria Fundación Jiménez Díaz (IIS-FJD, UAM), Madrid, Spain; 5 ILD Unit, Pneumatology Department, University Hospital of Bellvitge, IDIBELL. University of Barcelona, Barcelona, Spain

**Keywords:** ***Terc***, Telomere biology disorders, Telomeres, Pulmonary fibrosis, Aplastic anemia, CAST/EiJ, Senescence, Cell biology, Molecular medicine

## Abstract

Telomeres are terminal protective chromosome structures. Genetic variants in genes coding for proteins required for telomere maintenance cause rare, life-threatening Telomere Biology Disorders (TBDs) such as dyskeratosis congenita, aplastic anemia or pulmonary fibrosis. The more frequently used mice strains have telomeres much longer than the human ones which question their use as in vivo models for TBDs. One mice model with shorter telomeres based on the CAST/EiJ mouse strain carrying a mutation in the *Terc* gene, coding for the telomerase RNA component, has been studied in comparison with C57BL/6J mice, carrying the same mutation and long telomeres. The possible alterations produced in lungs and the haematopoietic system, frequently affected in TBD patients, were determined at different ages of the mice. Homozygous mutant mice presented a very shortened life span, more notorious in the short-telomeres CAST/EiJ strain. The lungs of mutant mice presented a transitory increase in fibrosis and a significant decrease in the relative amount of the alveolar epithelial type 2 cells from six months of age. This decrease was larger in mutant homozygous animals but was also observed in heterozygous animals. On the contrary the expression of the senescence-related protein P21 increased from six months of age in mutant mice of both strains. The analysis of the haematopoietic system indicated a decrease in the number of megakaryocyte-erythroid progenitors in homozygous mutants and an increase in the clonogenic potential of bone marrow and LSK cells. Bone marrow cells from homozygous mutant animals presented decreasing in vitro expansion capacity. The alterations observed are compatible with precocious ageing of lung alveolar cells and the bone marrow cells that correlate with the alterations observed in TBD patients. The alterations seem to be more related to the genotype of the animals that to the basal telomere length of the strains although they are more pronounced in the short-telomere CAST/EiJ-derived strain than in C57BL/6J animals. Therefore, both animal models, at ages over 6–8 months, could represent valuable and convenient models for the study of TBDs and for the assay of new therapeutic products.

## Background

Telomere biology disorders (TBDs), also known as short telomere syndromes or telomeropathies, compose a group of human rare, life-threatening diseases characterized by excessive telomere shortening^1,2^. Among these diseases are dyskeratosis congenita, aplastic anemia or pulmonary fibrosis. Telomeres are the terminal part of the chromosomes and are composed by tandem repetitions of the TTAGGG hexanucleotide bound by proteins of the shelterin complex^3^. Telomeres cannot be completely synthesized during DNA replication and their maintenance requires the reverse transcriptase activity of the telomerase complex^4^. Mutations in the genes coding for protein involved in telomere protection or elongation are causative of TBDs^5^.

In the absence of telomerase activity, progressive telomere shortening occurs during cellular proliferation and eventually drives to cell cycle arrest and subsequently result in cellular apoptosis or senescence^6^. In TBD patients this limited proliferative capacity can affect the renewal capacity of highly proliferative tissues such as the bone marrow or epithelia causing the bone marrow failure and skin manifestation of early onset TBDs such as dyskeratosis congenita or aplastic anemia^7^. On the contrary, the more frequent TBD, pulmonary fibrosis, is characterized by progressive respiratory failure at the adult age in the absence of skin manifestations or bone marrow failure. The main pathological observation is the altered structure of some pulmonary regions where alveoli show decreased epithelial cells and increased extracellular structures including myofibroblasts and fibrotic fibers^8^. In this case, the impaired renewal capacity of stem alveolar epithelial cells in response to tissue damage and their senescence, due to telomere shortening could be part of the pathological mechanism^9^.

TBDs are life-threatening diseases with poor prognosis and no curative treatment at the present time^10,11^. Only organ transplantation can significantly improve patient’s survival. Because of this reason there is an urgent need for the development of new treatments. However, this search is limited by the lack of good experimental models for the study of the biology of TBDs and the assay of new drugs. Laboratory mice show telomeres much larger than the human ones^12^ and, probably because of this reason, mice mutant for genes that code for the reverse transcriptase (*Tert*) and RNA (*Terc*) components of the telomerase do not show the manifestations of human TBDs caused by mutations in these same genes. Only after inbreeding for more than five generations mutant mice show decreased viability and fertility but not the usual manifestations of human TBDs^13,14^. Pulmonary fibrosis can be induced in mice by intratracheal instillation of the DNA-damaging antibiotic bleomycin. This treatment induces pulmonary fibrosis that is resolved in young animals a few weeks after drug administration, which differs from the evolution of fibrosis in TBD patients limiting the utility of this treatment as a model for the disease^15^.

One mice strain with a telomere size similar to the human one, CAST/EiJ, has been used previously as a model for TBDs^16,17^. The authors reported that *Terc*^−/−^ homozygous animals and *Terc*^+/−^heterozygous animals intercrossed for several generations showed decreased longevity and fertility. These animals presented tissue renewal defects reflected in hypocellular seminiferous tubules, intestinal lesions and hematopoietic dysfunction^18^. Bone marrow cells were more sensitive to chemotherapy and presented decreased reconstitution capacity. Some of these phenotypic characteristics resemble those of TBD patients and were more related to the length of the telomeres than to the genotype of each animal^17,18^.

The most frequent TBD, pulmonary fibrosis is presented either in relatives (familiar pulmonary fibrosis) or in unrelated patients (idiopathic pulmonary fibrosis), as previously mentioned. Bone marrow failure is also frequent in TBD patients and is often cause of their decease^7^. Because of these reasons the possible pulmonary and hematopoietic defects present in *Terc*-mutants were studied in this article. CAST/EiJ *Terc*^+/−^ animals were generated from the described C57BL/6J mutant strain. Heterozygous animals were intercrossed to simulate the familial form of the disease with dominant transmission. Wild type, heterozygous (*Terc*^+/−^) and homozygous (*Terc*^−/−^) animals were generated from these crosses. The characteristics of the lungs and the hematopoietic system were determined at different ages of the animals. The results obtained showed that this animal model reproduced some of the manifestations found in TBD patients including premature ageing of progenitor cells at both the alveolar epithelium and bone marrow. This alteration resulted in the loss of lung alveolar structure and reduced capacity of in vitro expansion of bone marrow cells at early ages in homozygous and heterozygous mutant animals.

## Methods

### Mice and animal procedures

Animal studies were made in accordance with European Community Directive and Spanish guidelines for the use of experimental animals and approved by the institutional committee of animal care and research (PROEX 223/19 from Comunidad de Madrid) and in accordance with ARRIVE guidelines. All animals were kept in a controlled environment (12/12 h light/dark cycle with lights on at 08:00, 21 ± 2 °C) with *ad libitum* access to food and water and closely monitored daily. Animals were sacrificed when signs typical of terminal illness appeared.

**Fig. 1:**
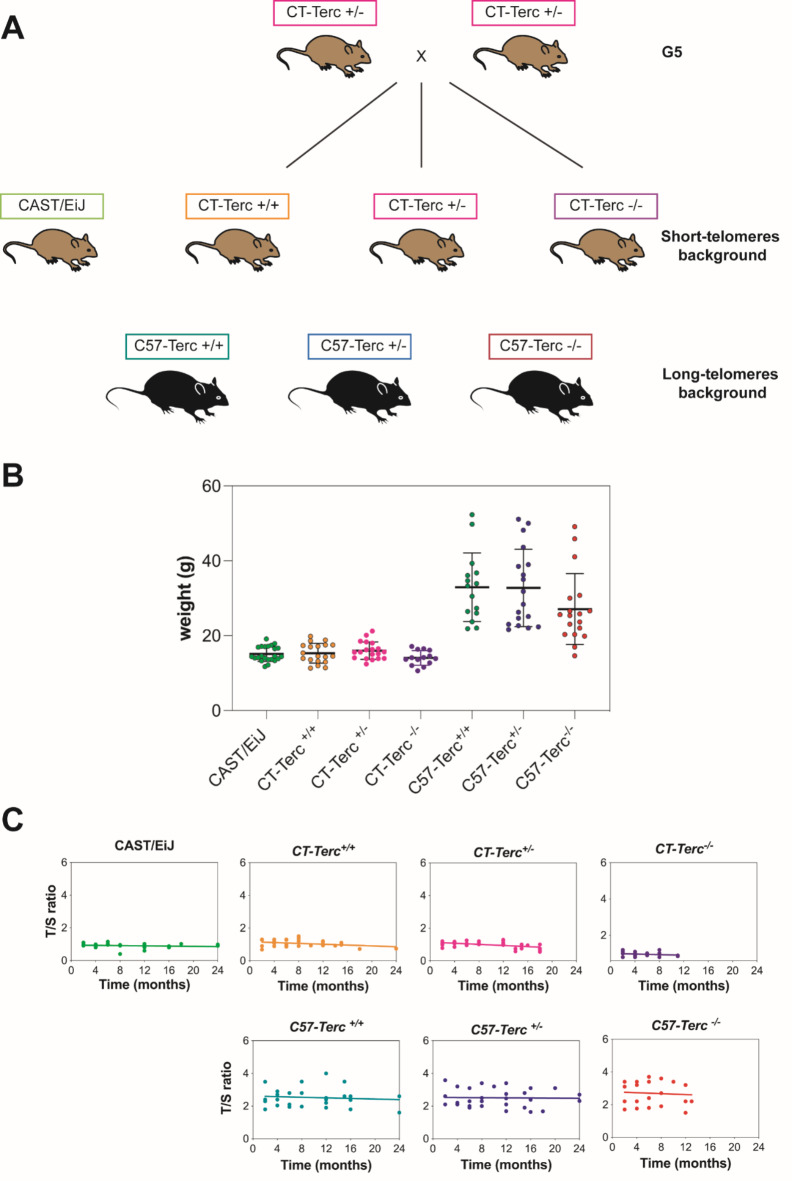
Generation of one strain of CAST/EiJ mice mutated in the *Terc* gene. **Panel A.** Breeding scheme and nomenclature of each generation. Heterozygous mice G5 were intercrossed to obtain G6: CT-*Terc*^*+/+*^, CT-*Terc*^*+/−*^ and CT-*Terc*^*−/−*^. In parallel, C57BL/6J *Terc*^*+/−*^ animals were crossed to obtain C57-Terc^*+/+*^, C57-Terc^*+/−*^ and C57-Terc^*−/−*^ animals. **Panel B**. Weight of the animals generated in the two genetic backgrounds (mice from 2 months to 10 months). **Panel C**. Telomere size (expressed as T/S ratio) of the mice strains with different *Terc* genotypes and genetic background at several ages (Time 0 to 24 months), determined from ear tissues. Linear regression analysis was used to assess the rate of telomere shortening with age. The Pearson correlation coefficient and linear regression equation were calculated (Y = −0,01723*X + 1,147) and were significant for CT-*Terc*^+/-^, p < 0.01.

CAST/EiJ and C57BL/6J *Terc*^*+/−*^ mice were purchased from Charles River Laboratories (Massachussets, USA). To generate mutant *Terc* mice on a short-telomere background as reported by Hao et al.^17^, we crossed CAST/EiJ females and C57BL/6J *Terc*^*−/−*^ males to generate heterozygous mice CAST/EiJ *Terc*^*+/−*^. Then, we backcrossed the hybrid animals onto the CAST/EiJ background for five generations (CT-*Terc*^*+/−*^ G1-G5). The CT*-Terc*^*+/−*^ G5 heterozygous mice were intercrossed to obtain CT-*Terc*^*+/+*^ G6, CT-*Terc*^*+/−*^ G6 and CT-*Terc*^*−/−*^ G6 (named CT-*Terc*^+/+^, CT-*Terc*^+/−^ and CT-*Terc*^−/−^). CT-*Terc*^*+/−*^ G6 mice were intercrossed to obtain the G7 generation and so on **(**Fig. 1A**).** In parallel, C57BL/6J *Terc*^*+/−*^ mice were intercrossed to obtain C57BL/6J *Terc*^*+/+*^, *Terc*^*+/−*^ and *Terc*^*−/−*^ animals (named as C57-*Terc*^*+/+*^, C57-*Terc*^*+/−*^ and C57-*Terc*^*−/−*^) for several generations. Both males and females were used in the experiments.

### Sample collection and processing

Mouse genomic DNA from ear tissue (punch at the set times) was isolated using the NZY Tissue gDNA isolation kit (NZYTECH) according to the manufacturer’s instructions.

Animals were euthanized using 10 µl/g of ketamine-xylazine. Lungs were perfused with 10% formalin, extracted and fixed in 10% buffered formalin. Fixed lungs were embedded in paraffin and cut into 5 μm sections for histopathological evaluation, immunohistochemistry (IHC) or immunofluorescence (IF) analysis. Hematopoietic organs (Bone Marrow, Peripheral Blood, spleen and thymus) were also isolated.

### Histopathological analyses, Immunofluorescence and immunohistochemistry

Hematoxylin and eosin (H&E) staining was performed for histopathological evaluation, and Masson´s trichrome staining served to evaluate collagen deposition. Lung tissue sections were stained using the standard hematoxylin-eosin (Sigma-Aldrich) (H&E) method. For Masson´s trichrome staining, samples were dewaxed and rehydrated through sequential xylene incubations and decreasing ethanol concentrations. Then, incubated in Bouin’s solution at 60ºC for 1 h and washed in running tap water for 5 min. Then stained in Weigert’s working hematoxylin (Merck) for 1 min, rinsed in running warm tap water for 10 min, and a last wash with distilled water. After were stained in Biebrich scarlet-acid fuchsin solution for 2 min, rinsed in distilled water, differentiated in phosphotunsgtic-phosphomolybdic acid solution for 15 min and 1% of acetic acid solution for 15 min and washed in distilled water. All preparations were dehydrated through 95% of ethyl alcohol, absolute ethyl alcohol, and cleared in xylene. Samples were analyzed under a light microscopy and images acquired using the Axiophot microscope (Zeiss), a direct microscope with transmitted light and epifluorescence (filters for DAPI, FITC and Red), using a DP70 (Olympus) color camera with the DP Controller image capture system. The objective used is a NEOFLUAR 40x objective. To quantify Masson’s trichrome staining, scanned images were processed with ImageJ software and analyzed to determine the collagen deposition.

To evaluate the pathological changes in the lung, mean linear intercept (MLI) was obtained from H&E stained sections according to established methods^19^. Ten sections at 20x magnification from each sample were chosen excluding areas with large airways and vessels. All images were acquired using an Axiophot microscope (Zeiss). To calculate MLI, five horizontal lines (400 μm length) per section were then used to count intercepts of alveolar septa.

Immunofluorescence (IF) was performed to assess fibrosis and expression of pro-surfactant protein C using mouse monoclonal anti-αSMA (1:200, SIGMA, A5228) and rabbit anti-pro-surfactant Protein-C (1:200, Millipore, AB3786) antibodies, respectively. For IF studies, the slides were first prepared as described above and antigen retrieval was performed using an unmasking buffer pH 9 (10 mM Tris-Base, 1 mM EDTA Solution, 0.05% Tween 20) in a microwave oven for 20 min. Sections were blocked with a Goat Serum blocking solution (5% Goat serum, 1% BSA, 0.2% Triton-X- 100 in PBS pH 7.1) for 45 min, and then, incubated with primary antibodies at 4ºC overnight in a humidity chamber. Sections were washed with PBS with 0.2% Triton-X-100 and incubated with the secondary antibodies for 60 min at room temperature. DAPI (D1306 Molecular Probes) or ToPro-3 (T3605 Molecular Probes) were used for DNA nuclear staining and slides were then mounted using ProLong Diamond antifade reagent (P36970 Molecular Probes). All images were acquired using a LSM710 (Zeiss) confocal microscope. All objectives were plan-apochromatic. Sequential scanning mode was used to avoid crosstalk between channels. All images correspond to a maximal projection and were processed with Zen2009 and ImageJ software for quantification. Quantification was performed determining the number of cells positive for a given antibody among the total number of cells of each field, and counting at least ten fields. Specific normalization is indicated in the legend of each figure.

Cellular senescence was evaluated by immunohistochemistry (IHC) using a rabbit antibody against p21 (CDKN1A) (1:200, Santa Cruz Biotechnology, sc-397). For immunohistochemical staining Antigen retrieval was performed by heating tissues section in 1X Dako Envision-Flex target retrieval Solution (Agilent) and endogenous peroxidase was removed with 3% H_2_O_2_ in methanol. Slides were blocked with normal goat serum (S-1000, Vector laboratories) and 0.1% Triton X-100 in PBS. The primary antibody was incubated at 4 °C overnight and the biotinylated secondary antibody (1:200, BA1000, Vector laboratories) at room temperature for 1 h. Then, we applied Standard Ultra-Sensitive ABC Staining Kit (32050, Thermo Fisher Scientific) and the sections were developed with 3,3‘-diaminobenzidine (D5637, Sigma) followed by nucleic counterstaining with hematoxylin. All images were acquired using an Axiophot microscope (Zeiss). Image J software was used for quantification of positive p21-cells (counting at least ten random visual fields for each slide).

### Telomere length measurements (TL)

Average telomere length was measured from total genomic DNA by a quantitative PCR assay, as previously reported^20,21^. For each PCR reaction, a standard curve was made by serial dilutions (4-fold per dilution) of genomic DNA, from a mouse F9 cell line extract, to produce five concentrations of DNA ranging from 8 to 0,125 ng/µl. A reference genomic DNA was also included from a mouse F9 cell line carrying the A353V mutation in the *Dkc1*gene as an internal control^22^. Samples were assayed by triplicate.

Forward and reverse telomeric primers used for amplification of telomeric DNA were 5’ CGG TTT GTT TGG GTT TGG GTT TGG GTT TGG GTT TGG GTT 3’ and 5’ GGC TTG CCT TAC CCT TAC CCT TAC CCT TAC CCT TAC CCT 3’ respectively. Primers for the reference control gene (mouse 36B4 single copy gene) were generated by Primer Express software (Applied Biosystems, Foster City, CA). Forward and reverse primers for the 36B4 portion of the assay were 5’ GTG CCA GCT CAG AAC ACT GG 3’ and 5’ TCA ATG GTG CCT CTG GAG ATT 3’, respectively. The final primer concentrations were 900 nM (telomere primers) and 300 nM (36B4 primers). PCR reactions were performed on the StepOnePlus™ Real-Time PCR System (ThermoFisher Scientific) in a final volume of 10 µL containing 3 ng of genomic DNA (sample and reference DNA), 1X PowerUp SYBR Green Master Mix (Applied Biosystem, Foster City, CA), and telomere or 36B4 primers. The thermal cycling profile for 36B4 was stage 1: 10 min at 95 °C; stage 2: 40 cycles of 15 s at 95 °C and 1 min at 58° with final acquisition. The thermal cycling profile for telomeres was stage 1: 10 min at 95 °C; stage 2: 40 cycles of 15 s at 95 °C, 30 s at 58° and 30 s at 72ºC with final acquisition. The telomere signal (T) was normalized to the signal from the 36B4 gene (S) to generate a T/S ratio indicative of relative telomere length.

### Characterization of the hematopoietic status in mice

Hematopoietic status of CT-*Terc*^+/+^, CT-*Terc*^+/−^, CT-*Terc*^−/−^, C57-*Terc*^+/+^, C57-*Terc*^+/−^ and C57-*Terc*^−/−^ mice strains was characterized by flow cytometry. Mice were euthanized at different time points (2, 6, 8, 10, 12, 18, 22 or 24 months) and hematopoietic organs (Bone Marrow (BM), Peripheral blood (PB), spleen and thymus were obtained to perform these analyses. First of all, approximately 750 µl of PB were extracted by cardiac puncture and mixed with 20 µl of EDTA while hind leg bones, spleen and thymus were maintained in PBS (Sigma-Aldrich). Later on, bones were flushed with 1 ml of PBS (2% BSA (Sigma-Aldrich) + 2% P/S) using 1 ml syringes (Novico Médica) and BD Microlance 3 0.5 mm x 16 mm (25G) needles (BD) to obtain the BM. BM was processed in a class II biosafety cabinet to keep the sterility of the sample taking into account the LSK cell sorting and the subsequent in vitro culture. Spleen and thymus were carefully disaggregated using 1 ml of PBS (2% BSA + 2% PS) and the same syringes with BD Microlance 3 1.2 mm x 40 mm (18G) needles (BD), first, and 0.5 × 16 mm needles, in the end. Hematologic counts of BM (only one femur and the whole BM separately), PB, spleen and thymus were analyzed using a XN-1000 V Haematology Analyzer (Sysmex). After that, all the samples were stained following the instructions detailed in Table 1. Red blood cells were lysed in the case of mature lineage and both LSK staining protocols. FMO controls were used in the LT-ST LSK staining.

**Table 1:**
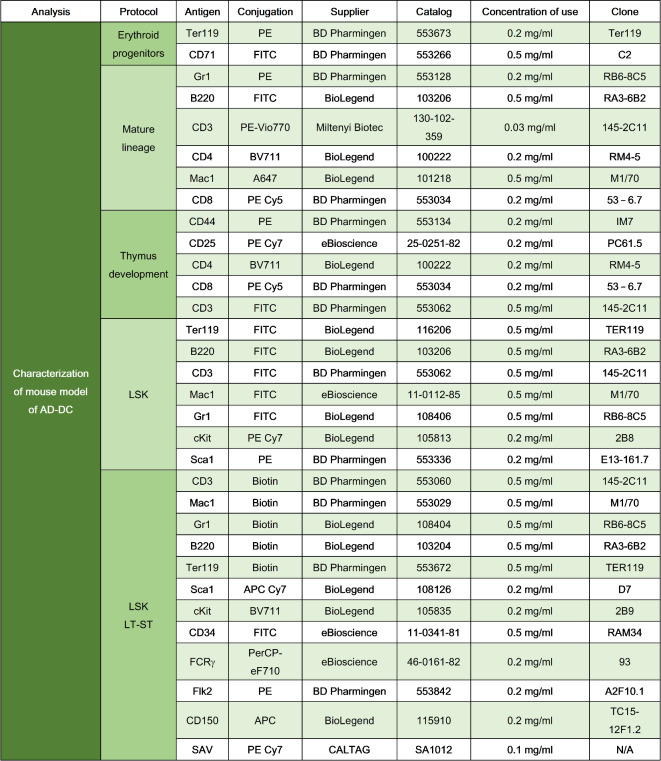
Summary of the different stainings conducted to characterize the hematopoietic status.

Fluorochrome abbreviations: phycoerythrin (PE), PE Cy5, phycoerythrin cyanine 7 (PE Cy7), phycoerythrin-violet 770 (PE-Vio770), peridinin chlorophyll protein complex-eFluor 710 (PerCP-eF710), Alexa Fluor 647 (A647), fluorescein isothiocyanate (FITC), allophycocyanin (APC), allophycocyanin cyanine 7 (APC Cy7) and brilliant violet 711 (BV711). Not applicable (N/A).

Moreover, LSK cells were sorted to study the in vitro expansion and the clonogenic potential. To do this, cellularity was determined in a XN-1000 V Hematology Analyzer (Sysmex) and samples were lysed using ammonium chloride lysis solution (0.155 mmol/l NH_4_Cl + 0.01 mmol/l KHCO_3_ + 10 − 4 mmol/l EDTA). Then, 1–8 × 10^7^ cells were stained with the cocktail of antibodies listed on Table 2 using DAPI (Thermo Fisher Scientific) at a concentration of 1 µg/ml as viability marker. This selection was performed by a BD Influx Cell Sorter (Becton Dickinson (BD). Once sorted, LSK cells were grown in StemSpam (StemCell Technologies) medium supplemented with 1% GlutaMAX (Gibco), 1% P/S solution (Gibco), 50 ng/ml mSCF (Novus Biologicals), 25 ng/ml FlT3L (EuroBioSciences) and hIL-6 (Novus Biologicals), 20 ng/ml hTPO (R&D Systems) and 10 ng/ml hIL-3 (Novus Biologicals) at 37ºC, normoxia, 5% CO_2_ and 90% relative humidity. Between 2 × 10^3^ and 2 × 10^5^ cells were cultured and maintained, when possible, at a concentration of 2 × 10^5^ cells/ml. LSK cells and total BM-MNCs were also seeded in methylcellulose-based medium MethoCult GF M3534 (StemCell Technologies) for 7 days at 37ºC, 5% CO_2_ and 90% relative humidity. Triplicates of 200 LSK cells or 2 × 10^4^ BM-MNCs were cultured in 1 ml of methylcellulose medium in 35 mm plates (Corning). After 7 days, total numbers of CFUs were quantified using an inverted microscope (Nikon).

**Table 2:**
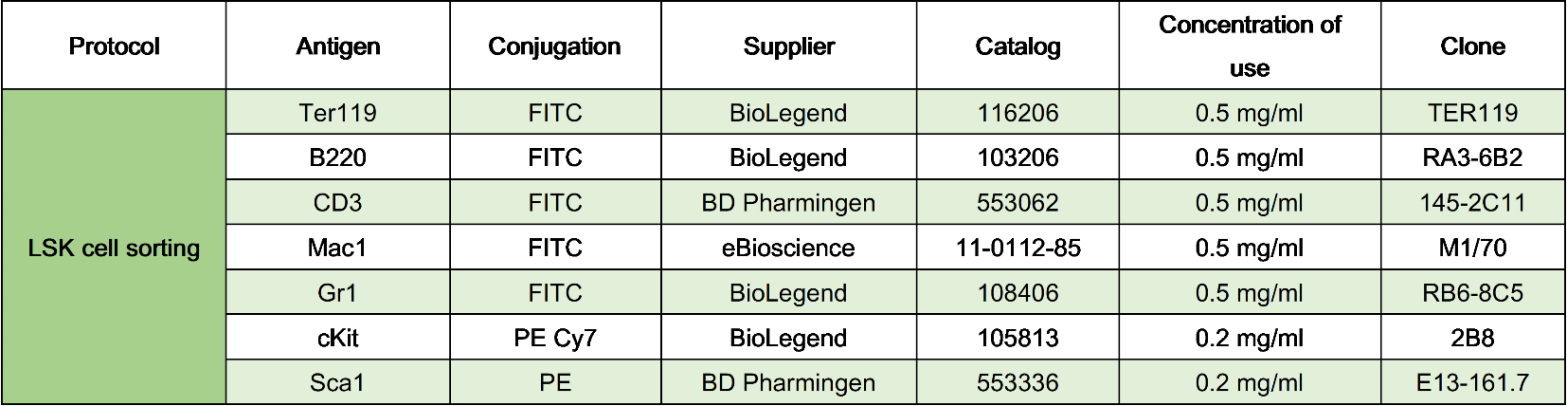
List of murine monoclonal antibodies used to perform the sorting of LSK cells.

### Statistical analysis

Statistical analyses were performed using GraphPad Prism version 9.0 (GraphPad software, United States). All the original data were showed as mean ± SD. Mann-Whitney test one-way ANOVA with post hoc Dunnett’s correction were applied. Survival was estimated using the Kaplan–Meier analysis. (log-rank test). Values of *p* < 0.05 were considered statistically significant.

## Results

### Generation of *Terc* mutant mice in the CAST/EiJ genetic background

Mutant *Terc* animals in the CAST/EiJ background were obtained from *Terc*^−/−^C57BL/6J animals^23^ by one initial cross between wild-type CAST/EiJ and mutant C57BL/6J animals. The mice obtained were backcrossed with CAST/EiJ wild type animals for four additional generations. The CAST/EiJ *Terc* mutated colony was maintained by crosses among heterozygous animals that allowed the generation of the three possible genotypes, *Terc*^+/+^, *Terc*^+/−^ and *Terc*^−/−^ animals. These animals have been named CT-*Terc*^+/+^, CT-*Terc*^+/−^ and CT-*Terc*^−/−^ along the manuscript. In parallel, heterozygous mice in the C57BL/6J genetic background were crossed to obtain the three possible genotypes that have been named C57-*Terc*^+/+^, C57-*Terc*^+/−^ and C57-*Terc*^−/−^ in the manuscript. These crosses are schematically shown in **Fig. 1A**.

Since C57BL/6J animals are larger than CAST/Eij animals, the weight of the different animals was determined to check the change in the genetic background and the possible impact of each genotype. The results are shown in **Fig. 1B**. CT animals grew to a similar weight as the CAST/EiJ parental strain and lower than that of the C57BL/6J animals. Interestingly we observed that homozygous *Terc*^−/−^ animals showed a tendency to be of smaller size that mice of the other genotypes but the difference, more noticeable in the C57BL/6J background, was not statistically significant.

The telomere length (TL) was determined in ear samples of animals of different genetic backgrounds and genotypes at several ages (Fig. 1**C).** The data indicated that TL in CT mice was similar to the CAST/EiJ strain and much shorter than the C57BL/6J strain as expected for the change in the genetic background of the animals. In addition, the size of telomeres in C57 animals was more variable between individuals than that of CAST/EiJ and CT mice. CT-*Terc*^*−/−*^ mice presented the shortest telomeres.

### Longevity of the *Terc* mutant animals

**Fig. 2:**
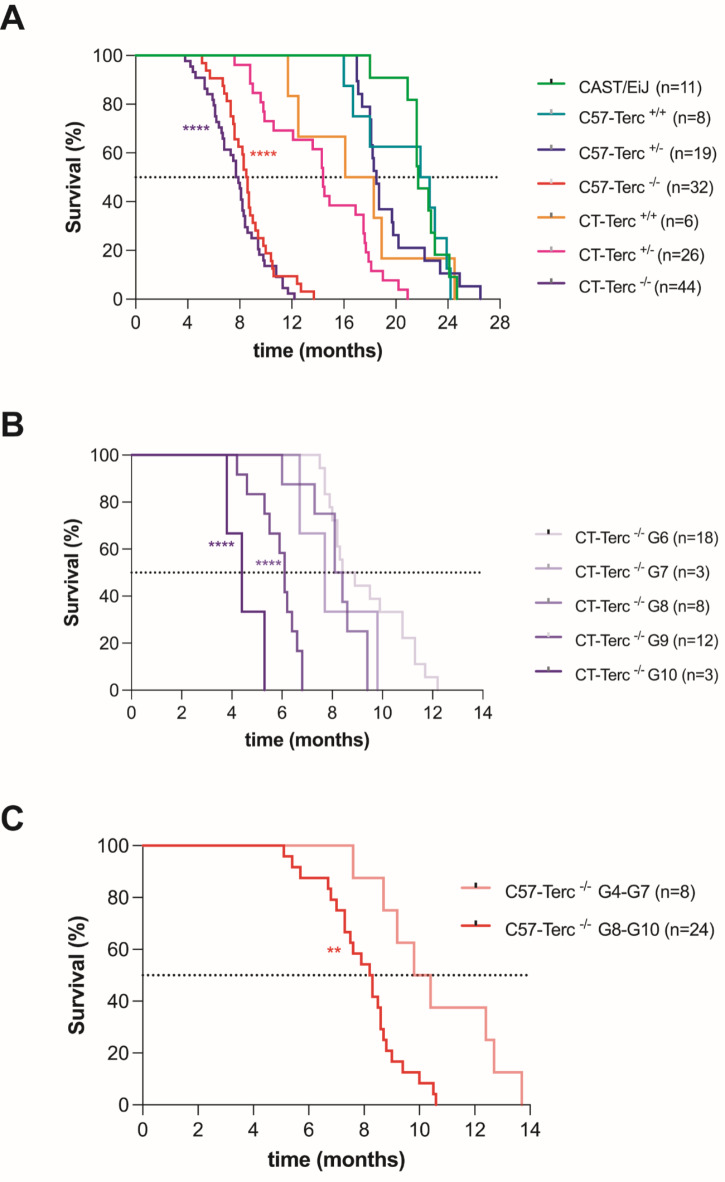
Life span of the mice from the different genotypes generated and their genetic background. **Panel A**. Kaplan-Meier analysis of overall survival of all the strains generated. Statistical significance was determined by Log-rank test. ****p < 0.0001 vs. wild-type animals. **Panels B and C.** Life span of the CT*-Terc*^*−/−*^ (B) and C57-*Terc*^*−/−*^ (C) mice obtained after crossing heterozygous animals for several generations (G6-G10). Statistical significance was determined by the Log-rank test. ***p < 0.001 (C57-*Terc*^−/−^ G8-G10 vs. C57-*Terc*^*−/−*^ G4-G7); ****p < 0.0001 (CT-*Terc*^−/−^ G9-G10 vs. CT-*Terc*^−/−^ G6).

The life span of the strains generated was determined, as shown in **Fig. 2A**. The most noteworthy observation was that homozygous mutant animals, in both genetic backgrounds, presented a life span significantly shorter than those of the other analyzed genotypes. The larger reduction was observed in CT-*Terc*^*−/−*^ mice with an overall survival of 8 months, compared to 18 months of the wild-type CT-*Terc*^*+/+*^ animals. C57-*Terc*^*−/−*^ mice also presented a very short life span of just 9 months on average. CT-*Terc*^*+/−*^ animals also showed a significant reduction in life span (15 months) although not as large as CT-*Terc*^*−/−*^ animals. Life span was similar for the other genotypes and for homozygous *Terc*^*+/+*^ and CAST/EiJ animals (19 to 24 months). Homozygous CT-*Terc*^*−/−*^ mice were obtained from crossing heterozygous animals during several generations and the life span of successive generations (G6 to G10) was also determined (Fig. 2**B**). The data obtained indicated that life span was reduced generation after generation and differences were statistically significant in G9 and G10 as compared to G6. The average life span was of little more than four months at G9/10. Homozygous C57-*Terc*^*−/−*^ mice also showed a decreased survival after four successive generations (from G4-G10) resulting in a median longevity of 8 months (Fig. 2**C**).

### Lung structure of CAST/EiJ and C57BL/6J mutant mice

**Fig. 3:**
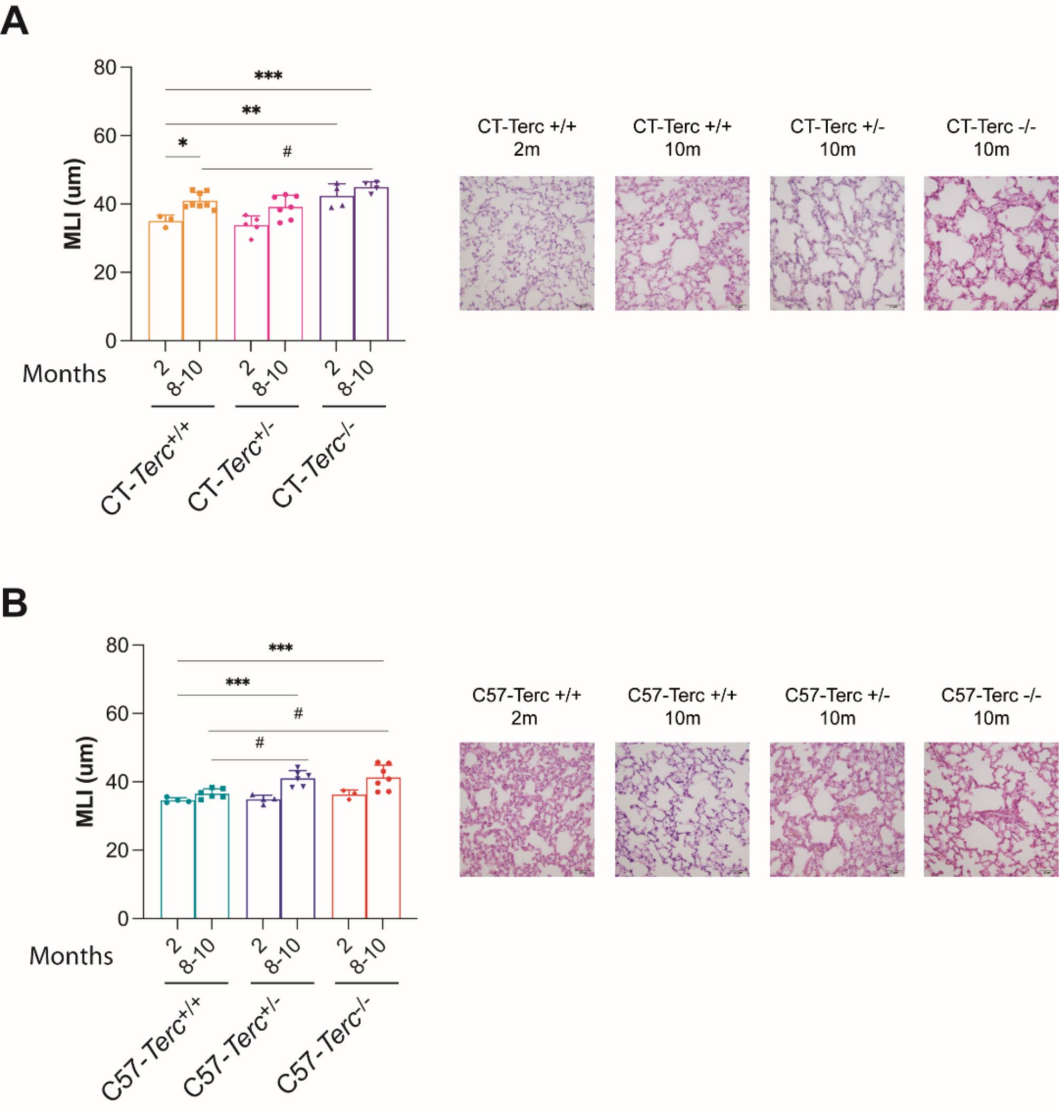
**Analysis on lung parenchyma structure in*Terc*** **mutant mice.** Histological sections of lungs were stained with Haematoxilin/eosine and the relative density of alveoli determined by the mean linear intercept (MLI) method. The median size of intercepts and SD are represented for lung preparations of CAST/EiJ (CT-, Left panel) or C57BL/6J (C57- right panel) mice. Magnification 20x. Lungs from two or 8–10 months-old mice were analyzed for each strain and genotype.

**Fig. 4:**
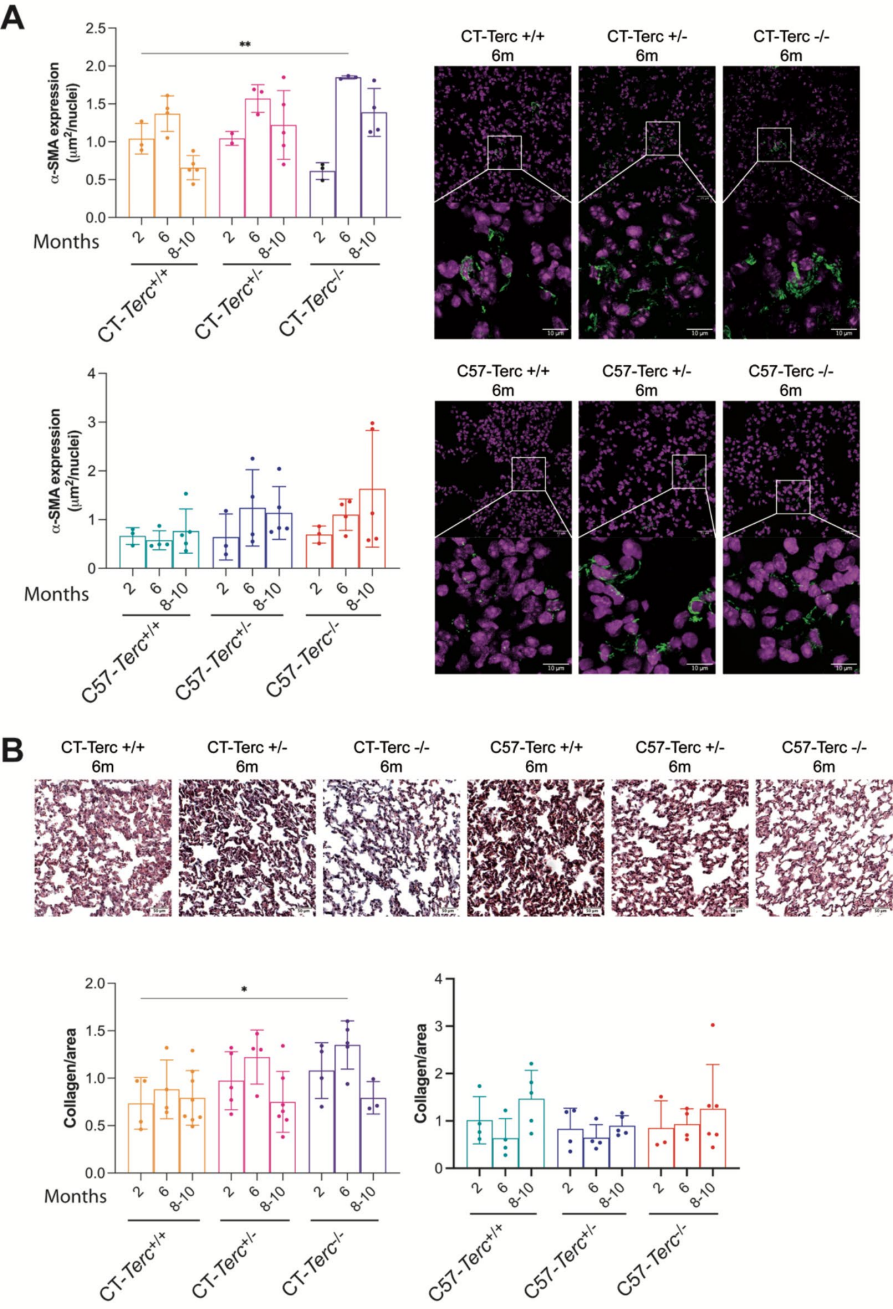
**Analysis of lung fibrosis in*Terc*** **mutant mice. PanelA**. Expression of αSMA in lungs of CT*-Terc* (**upper panel**) and C57-*Terc* (**lower panel**) mutant mice analyzed by immunofluorescence and quantified by image analysis (Left panels). Representative images of αSMA expression are shown at the right panels. The green signal showed the αSMA protein expression and the pink signals showed cell nuclei stained by TO-PRO-3. Data are presented as mean ± SD. **p < 0.01 vs. CT*-Terc*^*+/+*^ −2 m (one-way ANOVA and Dunnett´s comparison test). Magnification 40x. **Panel B**. Determination of lung fibrosis by Masson’s trichrome staining. Upper panel, representative photographs of lung sections stained with Masson trichrome, collagen type I fibers appear blue, muscle fibers and cytoplasm appear red and the nuclei appear blue-black. Magnification 40x. Lower panels, quantification of lung collagen deposition in CT*-Terc* (left panel) and C57-*Terc* (right panel) mice. Data are presented as mean ± SD and analyzed by ANOVA and Dunnett´s comparison test. *p < 0.05 vs. CT*-Terc*^*+/+*^ 2 m animals.

The most frequent pathological manifestation in patients with telomeropathies is the development of pulmonary fibrosis frequently inherited in more than one relative within the patient´s family. Because of this reason, the possible development of lung fibrosis and changes in lung structure were analyzed. Lungs were isolated from CAST/EiJ, and C57BL/6J mice with the three possible *Terc* genotypes at different ages. Initial observation of the HE stained samples indicated enlarged and more heterogeneous alveolar spaces in lungs from the mutant animals. To test this observation, morphometric analysis of the alveolar size was performed at 2 and 8–10 months of age by determining the mean linear intercept (MLI) (**Fig. 3**). An increase in MLI with the age was observed that is consistent with the observed loss of tissue integrity, with increased air space and reduced numbers of alveoli. This significant increase in MLI, at 8–10 months, was more noticeable in homozygous *Terc*^*−/−*^ mice in the two genetic backgrounds (Fig. 3) compared with wild type *Terc*^*+/+*^ mice. A similar increase was observed in heterozygous mice with statistical differences in 8–10-months-old *C57-Terc*^*+/−*^ animals. It would be interesting to determine telomere size and heterogeneity in the lung of these animals but PCR analysis does not provide this information on the variation of telomeres and alternative techniques would be required. The possible existence of lung fibrosis was determined next. The presence of myofibroblasts as biomarkers of fibrosis was analyzed by immunofluorescence using antibodies against alpha smooth-muscle actin (αSMA), as shown in **Fig. 4A**. Heterozygous and homozygous CT-*Terc*^−/−^ mice showed increased αSMA expression in comparison to CT-*Terc*^*+/+*^ mice that increased with age at 6 months in CT-*Terc*^+/−^ and CT-*Terc*^−/−^ and continue increasing at older age only in CT-*Terc*^−/−^ (Fig. 4**A**,** upper panel**). In C57 mice, maximal expression was observed at six months of age and decreased progressively at eight and ten months of age. In the case of C57 mice, αSMA expression was also higher in the *Terc*^*+/−*^ and *Terc*^*−/−*^ genotypes than in wild-type mice but differences were not statistically significant (Fig. 4**A lower panel**). Fibrosis at lungs was also determined by the Masson’s trichrome staining to visualize collagen type I fibers and the results are shown in **Fig. 4B**. Most of the data obtained were in agreement with the one shown above for αSMA expression. CT mice of the three genotypes showed maximal staining at six months of age that decreased at eight and ten months of age. CT-*Terc*^*−/−*^ mice showed higher staining at two months than the other two genotypes and also decreased at eight and ten months of age (Fig. 4**B**,** left panel**). In the case of C57-*Terc*^*+/+*^ and C57-*Terc*^*+/−*^ mice the staining was more similar between two and eight-ten months of age (Fig. 4**B right panel**) than that detected in the case of αSMA expression.

### Analysis of the presence of type two alveolar epithelial cells

**Fig. 5:**
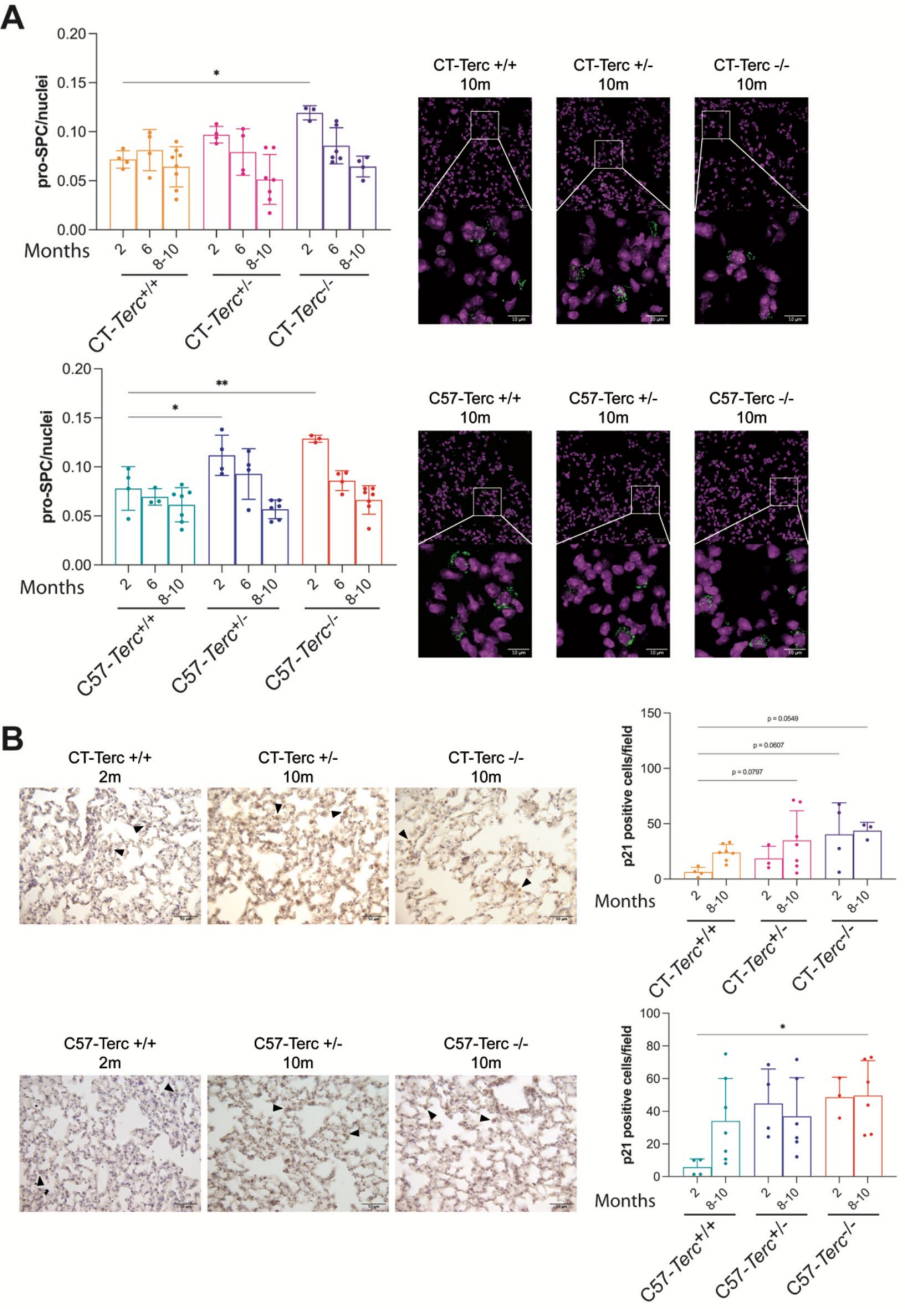
**Analysis of alveolar type 2 cells in the lung of*Terc*** **mutant mice at different ages. Panel A**. Quantification of alveolar type 2 cells in lungs from the different mice strains and genotypes. The number of Pro-SPC cells was normalized to the number of nuclei in each field. Right panels, representative photographs of lung sections. The green signal showed the pro-SPC protein expression and the pink signals showed cell nuclei stained by TO-PRO-3. Magnification 40x. Quantification of the presence of alveolar type 2 cells in CT*-Terc* (**upper panel**) and C57-*Terc* (**lower panel**) mice. **Panel B**: Immunohistochemistry analysis for p21 expression in lung sections from CT*-Terc* and C57-*Terc* mice at 2 and 10 months of age. Representative images (**left panels**) and quantification (**right panels**) of p21 positive cells. Magnification 40x. Arrowhead point to p21-positive cells (brown color). Quantifications were performed on ten random, different areas of each preparation. Data are presented as mean ± SD and analyzed by ANOVA and Dunnett´s comparison test. *p < 0.05 and **p < 0.01 (vs. wild type mice at 2 months of age).

The data presented above indicated that the oldest animals, even those of *Terc*^*−/−*^ genotype that had a very limited life span, did not show increased lung fibrosis with respect to early ages. Alternatively, disruption of the cellular homeostasis could also result in dysfunctional lungs that could affect survival of the animals. Type 2 Alveolar Epithelial cells (AEC2) are located in the alveolar surface and their main function is the secretion of surfactant to prevent alveolar collapse. In addition, they have been described as precursors of type 1 Alveolar epithelial cells I (AEC1), main components of the alveolar epithelium and involved in gas exchange. Because of these reasons, the number of AEC2 cells was determined in the lungs of the different mice strains and genotypes. Expression of the Pro-surfactant Protein C (Pro-SPC) was used as marker of AEC2 cells in immunofluorescence analysis of lung preparations (**Fig**. 5**A**). The data obtained for CAST/EiJ-derived mice indicate that the relative number of AEC2 cells significantly decreased with the age of CT-*Terc*^*+/−*^ and CT-*Terc*^*−/−*^ mice but not in wild-type mice (CT-*Terc*^*+/+*^) mice, as shown in **Fig. 5A**,** upper panel**. Similar results were obtained for *C57*BL/6J mice where a decrease in the relative amount of AEC2 cells was observed in the C57-*Terc*^*+/−*^ and C57-*Terc*^*−/−*^ genotypes (Fig. 5**A**,** lower panel**). It is noticeable that the expression of AEC2-marker cells is higher in mutant, *Terc*^*+/−*^ and *Terc*^*−/−*^ than in wild type *Terc*^*+/+*^ animals at two months of age in both genetic backgrounds. The larger amount of AEC2 cells might indicate an increased regenerative activity in the lung alveoli of young mutant animals.

### Lung senescence

In order to confirm whether the observed decrease on the AEC2 cell number was accompanied by an impaired epithelial cell cycle progression, we performed immunostaining of the senescence-associated p21 marker in lung sections from CAST/EiJ and C57BL/6J-derived mice (Fig. 5**B**). We examined senescence and found an increase in the number of p21 positive cells with the age in CT mice. This tendency seems to be more noticeable in CT-*Terc*^*−/−*^ mice even at early age (2 months) and statistically significant difference at eight months of age was found (Fig. 5**B**,** upper panels**). The same trend was observed for p21 expression in C57-*Terc*^*+/+*^ mice with the age. Interestingly, heterozygous and homozygous C57-*Terc*^*+/−*^*and* C57-*Terc*^*−/−*^ mice showed this increase as early as 2 months of age with statistical differences in C57-*Terc*^*−/−*^ mice at 2 and 10 months of age compared to wild type mice (Fig. 5**B**,** lower panel**). The amount of p21 positive cells showed a tendency to decrease between two and eight-ten month of age in C57-*Terc*^*+/−*^ animals which might be due to the more efficient elimination of senescent cells from early age in this strain.

### Haematological characterization of the mutant mice

**Fig. 6:**
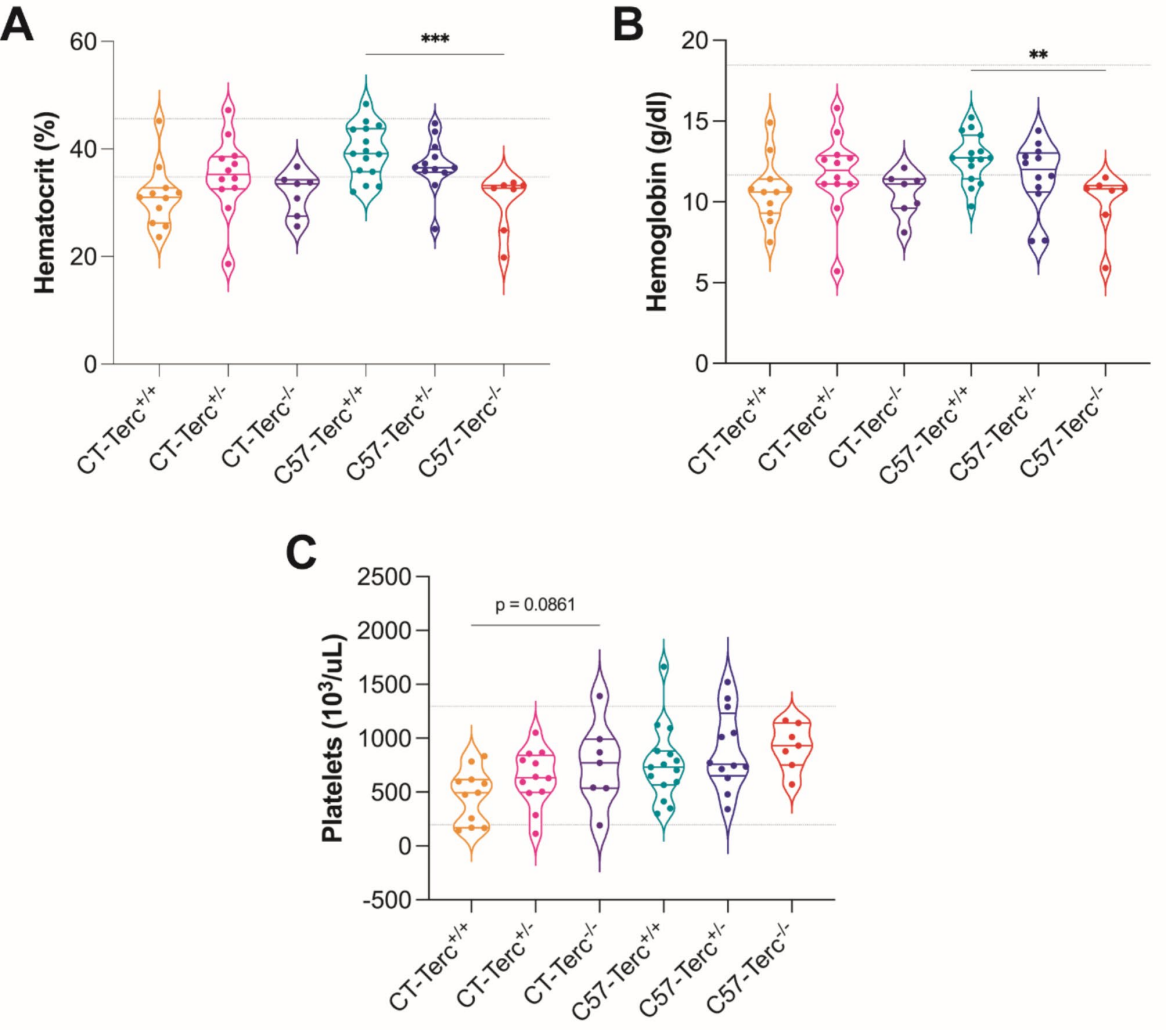
**Erythroid lineage and platelet counts in peripheral blood of the different mouse strains. (A)** Hematocrit (%). **(B)** Hemoglobin (g/dl). **(C)** Platelets (10^3^/µl). Normal ranges are defined by the gray line. Data were obtained from animals of the different ages analyzed in each strain and are expressed as mean ± SD analyzed by ANOVA and Dunnett´s comparison test. **p < 0.01 and ***p < 0.001 vs. wild type animals.

**Fig. 7:**
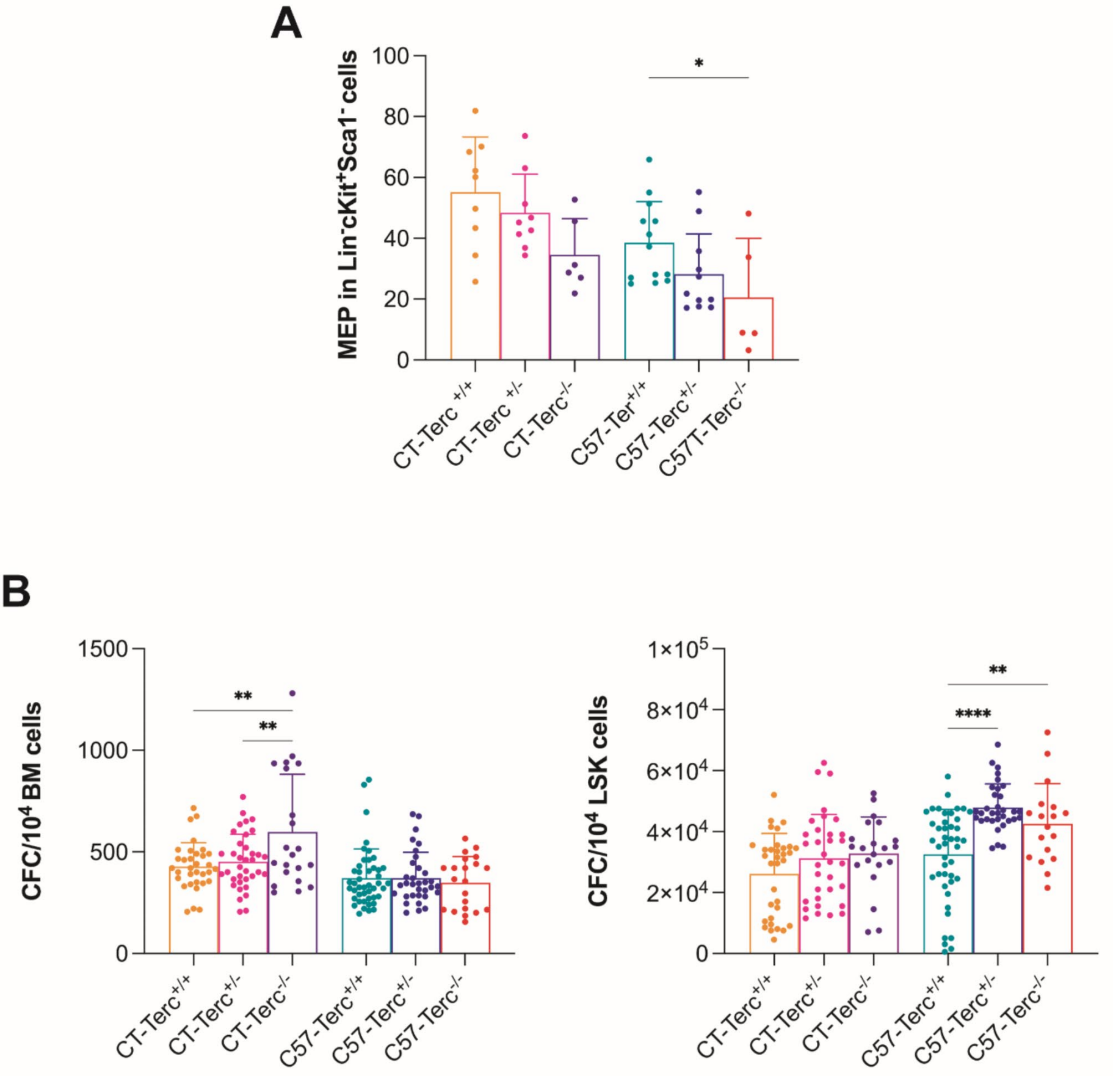
**Analysis of hematopoietic progenitor cells in*Terc*** **mutant mice. Panel (A)** Flow cytometry analysis of the megakaryocyte-erythroid progenitors. This quantification was assessed within the Lin^−^Sca1^−^cKit^+^ population (*n* = 65–138). **Panel (B)** Clonogenic potential studies in the characterized mouse strains. **Left panel**, CFCs per 10^4^ BM cells (*n* = 21–45). **Right panel**, CFC numbers per 10^4^ LSK cells (*n* = 18–43). Methylcellulose cultures were maintained for 7 days and CFC numbers are expressed as mean ± SD per ten thousand cells. Data were obtained from animals of the different ages analyzed in each strain and are expressed as mean ± SD. *p < 0.05, **p < 0.01, ***p < 0.001 and ****p < 0.0001 vs. wild type mice by ANOVA and Dunnett´s comparison test.

Telomeropathy patients often present alterations in the haematological system, including anemia that represent an important health burden. Because of this reason the main parameters of peripheral blood, spleen, thymus and bone marrow cells were determined. The cellularity in these organs was significantly lower in CASTEiJ than in C57BL/6J mice probably due to their smaller size but no difference was observed between genotypes in any of the two strains. Analyses of peripheral blood cells indicated no differences between genotypes in white blood cells (WBCs) or red blood cells (RBCs). Significant decreases were observed in the hematocrit and hemoglobin content in C57BL/6J mice where these parameters decreased in C57-*Terc*^+/−^ and C57-*Terc*^−/−^ animals in comparison to control mice (Fig. 6 **A**,** B**). In contrast, platelet numbers tended to increase in correlation with the severity of the mutation in both strains (Fig. 6**C**). Bone marrow multipotent progenitors were studied by flow-cytometry and significant differences were found in megakaryocyte-erythroid progenitors (MEPs) that represented significantly higher percentages in the CAST/EiJ animals that in the C57BL/6J ones. This increase was observed for the three genotypes although the percentage presented a tendency to decrease in *Terc*^*+/−*^ and *Terc*^*−/−*^ animals in both strains (Fig. 7**A**).

**Fig. 8:**
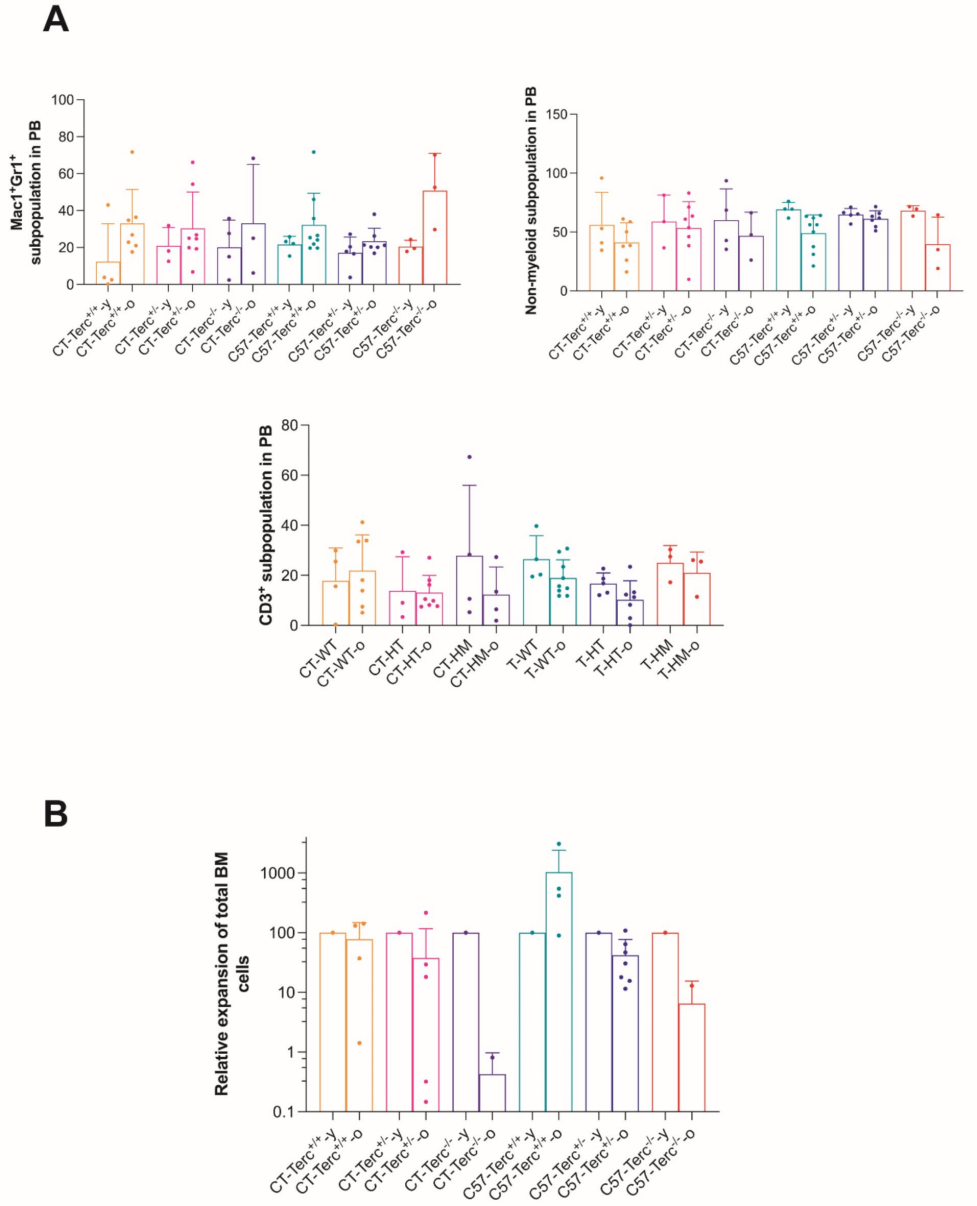
**Peripheral blood populations and expansion capacity of Bone Marrow cells from*Terc*mutant mice. Panel (A)** Upper left, levels of Mac1^+^Gr1^+^ cells (*n* = 3–9). Upper right, Percentage of Mac1^−^Gr1^−^ (non-myeloid) cells (*n* = 3–9). Lower panel, CD3^+^ cells within the non-myeloid subpopulation (*n* = 3–9). **Panel (B)** Relative in vitro fold expansion of old mice bone marrow total cells. 2 × 10^5^ cells were plated and the liquid cultures were maintained at a concentration of 5 × 10^5^ cells until their exhaustion (*n* = 2–7). These values are expressed as the relative increase or decrease as percentage of old mice expansion compared to young mice. Data are expressed as mean ± SD and analyzed by ANOVA and Dunnett´s comparison test.

The clonogenic potential was analyzed for BM and LSK cells (Fig. 7**B**,** left panel**). Significantly higher potential was observed for CT BM cells, as compared to C57 BM cells. The potential was higher for CT-*Terc*^−/−^ cells than in the other two genotypes. In contrast, the clonogenic potential of LSK cells was higher for C57 cells and was similar for the three genotypes analyzed (Fig. 7**B**,** right panel**). Subsequent studies were focused on the potential worsening of the haematological parameters in aged mice. In these studies the animals were divided in two groups: 2–6 months old, considered as young and 8–24 months old, considered as old. Analyses of myeloid subpopulations in peripheral blood revealed an age-related increase in the Mac1^+^Gr^+^ population and a decrease in the non-myeloid population Mac1^−^Gr1^−^ and CD3^+^ cells. These tendencies were more marked in *Terc*^−/−^ animals in both the C57BL/6 and CAST/EiJ genetic backgrounds (Fig. 8**A**). The analyses of in vitro expansion of BM cells indicated that their proliferative potential decreased in *Terc*^−/−^ animals and the effect was greater in the CAST/EiJ genetic background (Fig. 8**B**). Altogether, these results indicate the synergistic effect of age and *Terc* genotype on subpopulations in the PB and in vitro expansion of BM cells and that these alterations are increased in *Terc* mutants.

## Discussion

One difficulty for the study of Telomeropathies is the absence of good animal models that strongly limits the development of innovative therapies. As mentioned in the introduction, one of the reasons is that the most common mice strains used in the laboratories present much longer telomeres than humans and more heterogeneous. However, Hemann et al. described a number of wild mice strains with telomeres of similar length and size distribution as humans^24^. Consequently, we have developed a new mouse model based on the previous work of Dr. Carol Greider’s group and using one of them, the CAST/EiJ mouse strain, characterized by the presence of telomeres of a size similar to the human ones^16,17^. In this study we crossed C57BL/6J mice carrying a *Terc* mutation with CAST/EiJ mice for five generations to generate a *Terc*mutant strain in the CAST/EiJ genetic background. Animals of this new strain were always crossed among themselves in heterozygosis to maintain the dominant mode of transmission observed in human patients. Therefore, the homozygous animals analyzed in this study came from heterozygous parents. Mutant homozygous animals were not crossed among them as made in previous studies to exacerbate the phenotype of the animals in order to better simulate the human disease. The characteristics of the different strains were analyzed at different ages, including old mice, because some of the more frequent TBDs, such as the idiopathic pulmonary fibrosis, have a late onset at the adult age in the patients. In comparison, animals of 2–4 months of age had been analyzed in previous studies of CAST/EiJ animals^17,18,25,26^.

*Terc*^−/−^ mutant homozygous animals presented a very reduced lifespan of less than 10 months in both strains, with a C57BL/6J or a CAST/EiJ genetic background although it was shorter in the short-telomere strain. These results are similar to those described by Hao et al. for CAST/EiJ *Terc*^−/−^mice. A possible effect of gene anticipation was observed since lifespan decreased in progressive generations of homozygous mice obtained from heterozygous crosses. This effect was also observed in the two genetic backgrounds analyzed although lifespan was more reduced in the CAST/EiJ strain. These results are similar to those previously reported for the CAST/EiJ strain^17^. The reduced longevity of *Terc*homozygous mutants in the C57BL/6J background has been also reported previously^23^. In addition, TERT homozygous mutants in the CAST/EiJ genetic background also showed significantly decreased longevity^27^. Heterozygous animals lived over 16 months and showed a tendency to have a shorter lifespan than wild-type animals although differences were not statistically significant. These results might indicate that at least half of the wild-type telomerase activity is required for long survival of the mice independently of the basal telomere length of the strain. The reason for the early mortality of *Terc*^*−/−*^animals could not be established. Previous studies have proposed that increased mortality could be due to the increased intestinal permeability of homozygous mutants that, together with immune defects would result in increased bacterial infections^18^.

The present study has been centred in the two systems more frequently affected in TBD patients, respiratory and the hematopoietic system. Liver alterations are also frequent in TBD patients and we analyzed the possible existence of liver fibrosis and also p21 expression in liver but no differences were observed between genotypes in any of the two genetic backgrounds and the studies were not continued. Idiopathic pulmonary fibrosis is the most frequent presentation of TBDs with an onset in adults at an average of 50–60 years of age. Although the pathogenic bases of the disease are not well established, many authors consider that the lung manifestation is initiated by the alteration and lack of proliferative capacity of the alveolar epithelial cells 1 and 2 (AEC1 and AEC2). In particular, AEC2 cells are responsible for surfactant proteins secretion and also have the capacity to proliferate and differentiate into AEC1 cells that are the main components of the alveolar epithelia and responsible for oxygen/CO_2_exchange^28^. In IPF patients many AEC2 cells enter into a senescence state and acquire a senescence-associated secretory phenotype (SASP) producing a large number of soluble molecules including cytokines, chemokines, matrix-remodelling proteases, extracellular matrix components and growth modulators^29^. These molecules are proposed to interact with other cellular components including fibroblasts and macrophages that get activated. Fibroblasts secrete additional extracellular matrix components contributing to the fibrosis characteristic of the lungs of IPF patients while macrophages contribute to the formation of an inflammatory environment^30^. The possible presence of these alterations was analyzed in the *Terc* mutant mice. The presence of differentiated fibroblasts was tested by the expression of alpha smooth-muscle actin (α-SMA). Increased α-SMA expression was observed at six months of age in CAST/EiJ background mice, either Wild-type or mutant for *Terc* (CT, Fig. 4A). However, the expression decreased by 8–10 months of age indicating a transient activation of fibroblasts differentiation, although it remained higher in CT-*Terc*^−/−^ animals. In C57BL/6 animals increased expression of α-SMA was observed at similar levels at 6 and 8–10 months of age in mutant heterozygous and homozygous animals (Fig. 4B). Extracellular matrix deposition was determined by the presence of collagen fibers using the Masson’s trichrome staining of lung sections. Only small increases in collagen deposition were observed (Fig. 4B). Some of the increases correlated with the differences observed in α-SMA expression like the small increase observed at 6 months of age in CT-*Terc*^+/−^ and ^−/−^ animals or the one appreciated in C57-*Terc*^−/−^ animals at 10 months of age. In general, lung fibrosis was not observed in *Terc* mutant animals of either strain except for a small, transient increase at six months of age in CAST/Eij mice and at 10 months of age in C57BL/6 animals. These results are in agreement with previous studies showing that lungs from *Terc*^−/−^mice presented a decreased collagen content^25^. Transient fibrosis has also been observed in mice models of bleomycin-induced pulmonary fibrosis where fibrosis is induced reaching maximal levels 1–2 weeks after treatments and being resolved in the next weeks. These results allow us to speculate that *Terc* mutant mice might retain the capacity to resolve the pulmonary fibrosis induced in the mutants by about 6 or 10 months of age, depending on the strain.

The presence of type 2 alveolar epithelial cells (AEC2) was determined next. A progressive decrease in the amount of AEC2 cells was observed in *Terc*^+/−^ and ^−/−^ animals between 2 and 10 months of age (Fig. 5 A, B). The decrease was similar in both mice strains, independently of the basal telomere length. A decrease in the total number of AEC2 cells was also previously described in *Terc*^−/−^C57BL/6J mice inbreeded for four generations at the age of 2 months^25^.

Several authors have described increased senescence of AEC2 cells in pulmonary fibrosis patients, which would result in a decrease in the number of these cells^31^, reviewed in^30^). Because of this possibility, the expression of the senescence inducing p21 gene was analyzed (Fig. 5B). The results obtained indicate that p21 expression levels increase with age in wild-type animals. However, p21 levels were already increased in two-month old animals in *Terc*^−/−^ mice of both strains and even in heterozygous animals in C57BL/6J mice (Fig. 5B). These data indicate premature increase of senescent cells that could be related to their decreased number of AEC2 cells in *Terc*mutant mice and that could drive to pulmonary fibrosis^32^. These results are in agreement with previous studies that have described the presence of senescent AEC2 cells in *Terc*^−/−^mice intercrossed by three^26^or four generations^25^and in bleomycin-induced interstitial lung disease^33^. Accordingly, Alder et al.^34^ described that AEC2 cells isolated from *Terc*^−/−^ animals presented decreased self-renewal capacity. Recently, Lipskaia et al.^35^ using an inducible *Tert* expression model described that p21 expression is highest in lung endothelial cells (EC) and that *Tert* expression prevented exhaustion of progenitor EC cells with age. p21 expressing EC cells are required to maintain capillarity density an alveolar morphology and their lost could also contribute to the phenotype of *Terc* mutant animals analyzed in this article.

The analysis of lung sections also indicated the disorganization of the tissue with increased alveolar space in *Terc* mutant animals in both strains. A similar morphological alteration was observed in intercrossed *Terc*^−/−^animals^25,36^. The characteristics observed of increased p21 expression, decreased population of AEC2 cells and altered alveolar morphology, are reminiscent of those observed in the lungs of aged animals (reviewed in^37^). These data could indicate a premature lung ageing in *Terc* mutant mice that would be independent of the basal telomere length of the strains studied in this article.

In the second part of the study the possible alterations produced in the haematological system were approached since bone marrow failure is a frequent cause of decease in TBD patients. There was a clear downward trend in hematocrit and hemoglobin in C57BL/6J and CAST/EiJ mice as the severity of the mutation increased (Fig. 6A, B). It has been recently published that lower hematocrit and hemoglobin are observed in old mice in contrast to young ones^38^. We detected higher number of platelets, always within the normal ranges, as the severity increased in both genetic backgrounds (Fig. 6C). This increase could also be considered a sign of premature aging^39^.

Some differences between the CASTE/iJ and C57BL/6J genetic backgrounds were observed as MEPs percentage and clonogenic potential are higher for CT BM cells. These differences could be due the characteristics of the BM regulation in each strain since they exist for the three genotypes analyzed. Only CT-*Terc*^*−/−*^animals showed increased potential with respect to the other two genotypes which might reflect a compensatory proliferation to counteract the genetic defect. In addition, mutant mice with increased anemia could have died and would be under-represented in the population analyzed. This possibility could better explain the discrepancy observed with the results obtained by Balakumaran et al. in TBD patients^40^. These authors studied the BM stromal cell population from TBD patients and found that they presented reduced clonogenicity, spontaneous differentiation in adipocytes and fibrotic cells and decreased production of hematopoietic factors, contributing to bone marrow failure.

We also observed diminished MEPs as the severity of the mutation increased in both genetic backgrounds (Fig. 7A). Taking into account that erythroid differentiation ability is the first to be lost in inherited bone marrow failures, an impairment in this potential might be the underlying cause of the accumulation in the MEP compartment in CAST/EiJ mice given that apparently hematopoietic stem cells were perfectly able to differentiate up to this step.

Decreases in Mac1^−^Gr1^−^ and increases in Mac1^+^Gr1^+^ populations, as observed in Fig. 7 more evidently in C57-*Terc*^−/−^ and CT-*Terc*^−/−^ groups, can be considered as further evidence of aging in these mice.

It is widely known that it is extremely difficult to mimic BMF in animal models. For example, *Fanca*^−/−^ mouse model does not reproduce the characteristic BMF of FA patients. In fact, thrombocytopenia is the only hematological problem that can be observed in *Fanca*^−/−^mice^41^. As well as has been published in *Terc*^−/−^mouse model^42^, clear BMF was not detected and alterations in spleen and thymus were not expected in our *Terc* mutant mouse model although premature death became evident (**Fig. 2**). Consequently, those mice that prematurely died might have developed BMF and we could be analyzing the surviving old mice.

Decreased expansion of aged BM cells was observed in both genetic backgrounds: C57BL/6J and CAST/EiJ mice (**Fig. 8A**). Growth capacity was not affected in C57-*Terc*^*+/+*^ and barely in CT-*Terc*^*+/+*^. Nevertheless, this impairment became worse as the severity of the mutation increased, that is to say, BM cells from *Terc*^*−/−*^ mice grew poorer than *Terc*^*+/−*^ mouse BM cells. Therefore, we demonstrated that the functionality of BM cells underwent stressful procedures, such as in vitro expansion, which was quite compromised by a synergistic effect of age and mutations in *Terc* (**Fig. 8B**). The results obtained on hematopeietic cells are in general agreement with those previously reported for CAST/EiJ *Terc*mutant mice showing hematopoietic and immune defects that resembled those present in dyskeratosis congenital patients^18^.

## Conclusions

The data reported are in agreement with a premature ageing at both lung alveoli and BM cells in *Terc* mutant mice. The phenotype is more manifest in *Terc*^−/−^ animals but is also observed in *Terc*^+/−^ heterozygous animals from 6 months of age. The alterations were observed in the CAST/EiJ-derived animals, with shorter telomeres, but also in C57BL/6J animals indicating that the genotype of the animals could be more important that the basal telomere length in this model. However, the presence of mutated *Terc* induces a progressive telomere shortening that also impact in the progression of the alterations, as shown by the decreased life span of homozygous mutant mice in subsequent generations of heterozygous inbreeding. Some of the alterations observed in these mice models have been described in TBD patients as the presence of AEC2 senescent cells, the disorganization of the alveolar structure or the limited expansion capacity of the bone marrow precursor cells. Therefore, these animal models, at ages over 6–8 months, could represent valuable models for the study of these diseases and for the assay of new therapeutic products, thus avoiding waiting until advanced ages to develop evaluable parameters in the analysis of therapeutic activity. In comparison to previously described models, the analysis of aged animals would avoid the generation of mutant animals interbred for 4–5 generations to observe a testable phenotype in younger animals.

## Data Availability

Data used and/or analyzed during the current study are available from the corresponding authors upon reasonable request.

## Abbreviations

AEC1: Alveolar Epithelial cell type 1
AEC2: Alveolar Epithelial cell type 2
BM: Bone Marrow
BMF: Bone Marrow Failure
HE: Hematoxylin and eosin
IF: Immunofluorescence
IHC: Immunohistochemistry
MEPs: Megakaryocyte erythroid progenitors
MLI: mean linear intercept
PB: Peripheral blood
Pro-SPC: Pro-surfactant Protein C
RBCs: Red Blood Cells
α-SMA: alpha smooth muscle actin
TBD: Telomere Biology Disorder
TL: telomere length
WBCs: White Blood Cells
